# Effects of Prebiotic and Synbiotic Supplementation on Glycaemia and Lipid Profile in Type 2 Diabetes: A Meta-Analysis of Randomized Controlled Trials

**DOI:** 10.15171/apb.2018.065

**Published:** 2018-11-29

**Authors:** Sepideh Mahboobi, Fatemeh Rahimi, Sadegh Jafarnejad

**Affiliations:** ^1^Department of Community Nutrition, Shiraz University of Medical Sciences, Shiraz, Iran.; ^2^Faculty of Public Health, Kermanshah University of Medical Science, Kermanshah, Iran.; ^3^Research Center for Biochemistry and Nutrition in Metabolic Diseases, Kashan University of Medical Sciences, Kashan, Iran.

**Keywords:** Prebiotic, Synbiotic, Glycemic Status, Lipid Profile, Diabetes, Meta-analysis

## Abstract

***Purpose:*** Diabetes Mellitus (T2DM) as a chronic disease, is on rise in parallel with other non-communicable diseases. Several studies have shown that probiotics and prebiotics might exert beneficial effects in chronic diseases including diabetes. Because of controversial results from different trials, the present study aims to assess the effects of prebiotic/synbiotic consumption on metabolic parameters in patients with type2 diabetes.

***Methods:*** A systematic literature search was performed on randomized controlled trial published in PubMed/Medline, SciVerse Scopus, Google scholar, SID and Magiran up to March 2018. Of a total number of 255 studies found in initial literature search, ten randomized controlled trials were included in the meta-analysis. The pooled mean net change were calculated in fasting blood-glucose [FBG], Hemoglobin A1c [HbA1c] and lipid markers (total cholesterol [TC], triglyceride [TG], low-density lipoprotein cholesterol [LDL-C], high density lipoprotein cholesterol [HDL-C]). The meta-analyses was conducted using Revman Software (v5.3).

***Results:*** The pooled estimate indicated a significant difference for the mean change in FBG, HbA1c and HDL in treatment group in comparison with control group. Subgroup analysis by intervention showed a significant difference in TG, LDL and HDL (synbiotic group) and in TG, TC, FBG, HDL and HbA1c (prebiotic group) compared with placebo. In another subgroup analysis, high quality studies showed significant reductions in TG, TC, FBG and HbA1c in intervention group compared with placebo group.

***Conclusion:*** In summary, diets supplemented with either prebiotics or synbiotics can result in improvements in lipid metabolism and glucose homeostasis in type 2 diabetic patients.

## Introduction

Type2 Diabetes Mellitus (T2DM) as a chronic disease, is on rise in parallel with other non-communicable diseases, not only in adults but also in children and adolescents worldwide.^1^ 190 million people were diabetic in 2008 and according to estimates this number will reach 366 million in 2030.^2^ Both host genetics and environmental factors are clearly associated with the onset of T2DM.^3^ Epidemiological studies revealed that there is a positive relation between high blood glucose levels (glycemia), lipid abnormalities and cardiovascular diseases.^4^

Beyond the generally acknowledged idea that genetic factors assume an imperative part in diabetes susceptibility, developing evidence has shown that some variables such as chemical and diet, can affect diabetes development. Increasing evidence indicates that gut microbiota is strongly associated with type2 diabetes development.^5^

As compared to non-diabetic subjects, diabetic subjects experienced a decrease in butyrate-producing bacteria such as Roseburia intestinalis and increases in Lactobacillus gasseri and some Clostridium microorganisms. Moreover, increased expression of microbiota genes involved in oxidative stress and inflammation was observed in diabetic patients.^6^

Probiotics, prebiotics and synbiotics may alter the gut microbiota and stabilize microbial communities. Probiotics are defined as live microorganisms that can exert health effects on the host when administered adequately and were first described by Metchinkoff in 1908.^3,7^ Probiotics have a pivotal role in the host’s general health.^3^ These products can be used as anti-diabetic agents since various studies have shown their possible ability to improve glucose homeostasis and delay the progression of diabetes in animal models.^8-11^

A prebiotic is non-digestible food component that selectively stimulates the activity or growth of a few number of probiotic bacteria in the colon, especially, but not exclusively, lactobacilli and bifidobacteria.^12^ Manipulation of gut microbiota through prebiotic consumption can exert metabolic health benefits in high risk individuals.^13^

Synbiotic is a combination of probiotics and prebiotics which promotes host’s metabolic health by selective growth stimulation and healthy microorganism activation. Synbiotic is a compound beyond a mixture of probiotics and prebiotics but there is a synergistic effects of these two components that makes it a more effective supplement compared with probiotic or prebiotic separately.^14^

Several studies suggest positive effects of synbiotics on blood lipid profile,^4,15,16^ while some other studies have failed to prove the positive effects of probiotics, as a part of synbiotics, on cholesterol.^17,18^ Furthermore, it has been observed that synbiotics might promote fasting blood glucose (FBG), insulin levels, and the homeostasis model assessment-insulin resistance (HOMA-IR).^15^

RCTs evaluating effects of prebiotics alone or in combination with probiotics have yielded controversial results. Therefore, there is a need for a study to provide a comprehensive conclusion on the effects of prebiotic/synbiotic supplementation in diabetic patients. The present study aims to evaluate whether prebiotic/synbiotic consumption can beneficially affect metabolic parameters including glycemic status and lipid profile in patients with type 2 diabetes in compared with non-diabetic subjects.

## Materials and Methods

The current meta-analysis was undertaken in accordance with Preferred Reporting Items for Systematic reviews and Meta-Analyses(PRISMA) statement for systematic review and interventional researches.^19^

### Data Sources and Search Strategies

Systematic research was conducted on the following electronic databases: PubMed/Medline®, SciVerse Scopus®, Google scholar, SID® and Magiran®; in order to detect the medical literatures for Randomized Controlled Trials (RCTs) of the effects of synbiotic and prebiotic supplementation on lipid profile and glycaemia in patients with DM. These databases were searched up to March 2018. Moreover, the keywords were applied included: (prebiotic OR synbiotic OR symbiotic OR fructooligosaccharide OR fructo-oligosaccharide OR galactooligosaccharide OR galacto-oligosaccharide OR inulin OR lactulose OR FOS OR GOS OR oligofructose) and (cholesterol OR “plasma lipids” OR triglycerides OR TG OR HDL-c OR LDL-c OR “serum lipids” OR FBS OR FBG OR “fasting blood glucose” OR HbA1c).

The search strategy was implemented based on the database orientations using Boolean operators (OR and AND), parenthesis and quotation marks. Quotation marks were used to search for exact terms or expressions; parenthesis was used for representing a group of search words or combination of two categories of search words to capacitate all probable combinations of statements.

### Study Selection

Studies must have had these following inclusion criteria to enter this meta-analysis: a controlled clinical trial in humans, that included synbiotic or prebiotic supplement intervention, in forms of either supplement or enriched food, and evaluated at least one of the following outcomes: TG,TC, LDL-c, HDL-c, FBG and HbA1c. In addition, only the human RCTs published in English or Persian language were used in the meta-analysis, whereas animal/molecular, observational, preclinical and duplicate studies, commentaries, case reports or series, conference proceedings, editorials, and book chapters/reviews were excluded.

### Data Extraction and Quality Assessment

Data were extracted from qualified papers by two independent authors (F.R and S.M) using predefined protocols and cross-checked. Any divergence of opinion was resolved by consulting a third reviewer (S.J). The following data were extracted from the selected articles: year of publication, region (country), sample size, age, sex, follow-up duration, design of study, distinguishing the type of consumed supplement (prebiotic, synbiotic or placebo), dose of consumed synbiotic and prebiotic, methods of synbiotic/prebiotic delivery, clinical condition, and mean changes of metabolic indices. All the above-mentioned data were arranged in the Microsoft Office Excel® 2013 document (Microsoft Corporation, Washington, USA).

The Jadad Scale was computed to assess the methodological quality of included clinical trial studies. Jadad Scores range from 0(very low) to 5(very high) based on 3 distinct parts of randomization, double blinding, and follow-up. This scale assigns 1 point for mentioning randomization in the text, 1 point for mentioning blinding in the text, 1 point for proper description of the fate of all subjects. 1 point if the randomization method was appropriate (−1 if inappropriate) and 1 point if the double-blinding was appropriate (−1 if inappropriate).^20^

### Quantitative data synthesis

The meta-analyses was conducted using Review Manager Software (Version 5.3; Oxford, England). Furthermore, metabolic factors alterations from the baseline to the final time point of RCTs were calculated as the Mean Differences (MD) with the 95% Confidence Interval (CIs).

All values were collated as in mg/dL and mmol/L. Mean net changes and standard deviation in metabolic indices including TC, TG, LDL-c, HDL-c, HbA1C and FBG were calculated for all studies. The conversion factor for cholesterol (consist of HDL-c, LDL-c and TC), TG and FBG was 1 mmol/L=38.66 mg/dL, 1 mmol/L=88.57 mg/dL and 1 mmol/dL=18 mg/dL; respectively.

For assessment the degree of inconsistency across studies by heterogeneity, the I2 statistic was used and either fixed or random effects models were used according to the findings. An I2 value of larger than 50% reflects moderate to high heterogeneity. To clarify the influence of studies characteristics, pre-specified subgroup analyses were conducted based on the Cochrane handbook. We assessed the publication bias by visual inspection of funnel plots test. Asymmetric shape of funnel-plot can be indicative of a publication bias. Moreover, Egger’s weighted regression test and Begg’s rank correlation test were used to examine possible bias. A P-value of less than 0.05 was considered as statistically significant.

## Results and Discussion

### Study selection

**Figure 1:**
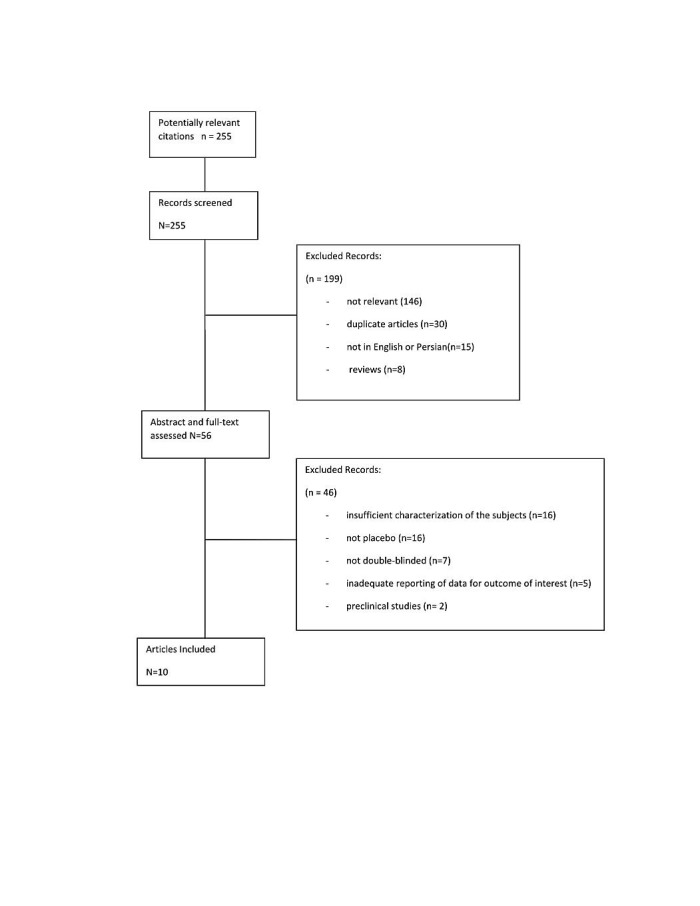
Meta-analysis Flow Diagram

A flow chart of literature search and selection is presented in Figure 1. In our initial search, 255 potentially relevant articles were identified. Of these, 8 were excluded because they were review articles. 15 were excluded because they were not available in either English or Persian language. Moreover, one-hundred forty six studies were excluded after screening the titles and summaries due to irrelevance and fifty-six potentially eligible articles were left for full-text assessing. Out of the 56 studies, 46 were excluded because they were preclinical studies or with lacking characterization of the subjects, with inadequate reporting of data, with insufficient data of placebo groups or with outcome measures other than lipid and glycemic indices. Finally a total of 10 RCTs were included in the present mete-analysis (Figure 1).

### Study characteristics/quality assessment

Table 1 shows characteristics of the included studies. These studies were all RCTs published up to March 2018. A total of 506 participants (including 251 subjects in the intervention group and 255 subjects in the control group) were reanalyzed in this study. The age of participants in trials varied from 20 to 70 years. Duration of intervention varied from 4 to 12 weeks. Four studies^4,15,16,21^ used the synbiotic and six studies^22-27^ used the prebiotic as intervention. Based on several previous meta-analysis studies which indicated the studies with Jadad score of more than 3 as high quality studies,^28-30^ seven studies were classified as high quality studies^16,21-25,27^ and the remaining three^4,15,26^ as low quality studies.

The present systematic review and meta-analysis summarizes data from 10 RCTs including a total number of 506 participants. Our finding supports the idea that prebiotic supplementation may improve some factors of blood lipids and glycemic control in type2 diabetic patients. In general, the findings are consistent with results of most individual studies; of 10 included studies, 8 reported some beneficial effects of prebiotic/synbiotics on glycaemia and lipid profile.^4,15,16,22-24,27,31^ In recent years, a considerable number of researches have been conducted with a focus on probable beneficial effects of prebiotics or synbiotics on metabolic profile in different target groups. There are limited systematic reviews which investigate the effects of synbiotic and/or prebiotic supplements on metabolic parameters in diabetic and/or overweight subjects. However, lack of subgroup analyses is considered as their limitation.^32,33^ Therefore, our study is the first comprehensive meta-analysis, evaluating whether synbiotic/prebiotic supplementation has favorable effects on metabolic indices on diabetic patients based on both intervention and study quality analyses.

### The effects of intervention on blood glucose and lipid concentration

Since there were different units for applied indices in included trials, they were transformed to single unit (mg/dl) for TG, TC, LDL-c, HDL-c and FBG. As there were significant heterogeneity among studies for the mean change of most indicators (except for HDL-c), the random effects model was used for pooling data.

**Figure 2:**
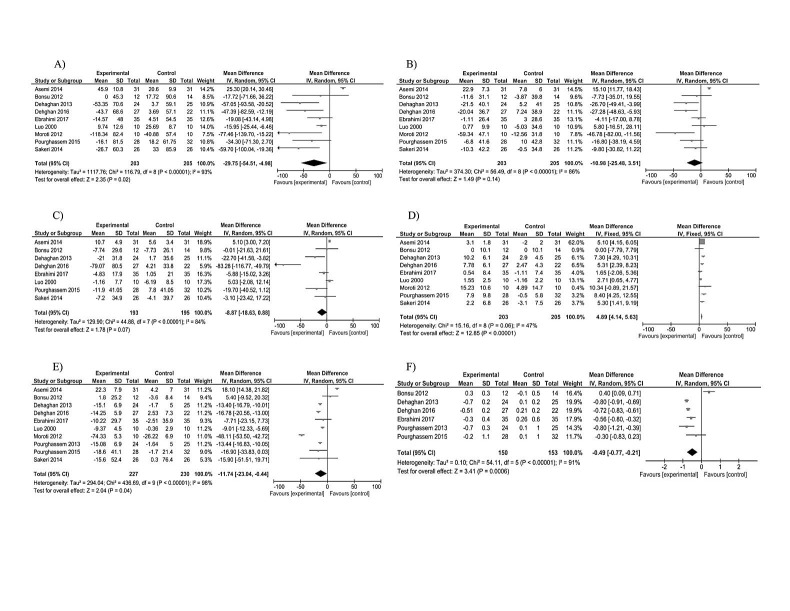
Forest plot showing the association between prebiotic/synbiotics and serum TG(A), TC(B), LDL-C(C), HDL-C(D), FBG(E), HbA1c(F). Abbreviations: TG, triglyceride; TC, total cholesterol; LDL-C, low-density lipoprotein cholesterol; HDL-C, high-density lipoprotein cholesterol; FBG, fasting blood glucose; d. Random effects model was used to pool the mean change of indicators.

The pooled mean net change for TG in treatment group was -29.75 compared with control group that was statistically significant [95%CI: -54.51, -4.98; p for heterogeneity < 0.00001, I2=93%]. For serum total cholesterol level, the pooled mean net change was -10.98 in treatment group [95%CI: -25.48, 3.51; p for heterogeneity < 0.00001, I2=86%] that did not differ significantly compared with control group. Included studies investigated effects of prebiotic supplementation on LDL-c levels. There was a marginally significant difference between pooled mean net change for treatment group compared with placebo group, considering this marker [WMD -8.87, 95%CI: -18.63, 0.88; p for heterogeneity < 0.00001, I2=84%]. Intervention group showed a significant rise in HDL-c compared with control group [WMD 4.89, 95%CI: 4.14, 5.63; p for heterogeneity =0.06, I2=47%] (Figure 2).

Se rum levels of FBG were measured in all included trials. The pooled estimate indicated a significant difference for the mean change in both FBG and HbA1c in treatment group in comparison with control group [FBG WMD -11.74, 95%CI: -23.04, -0.44; p for heterogeneity < 0.00001, I2=98%]. Mean change for HbA1c was calculated in six included studies. Total mean difference for HbA1c was -0.49 [95%CI: -0.77, -0.21; p for heterogeneity < 0.00001, I2=91%] (Figure 2).

### Publication bias

**Figure 3:**
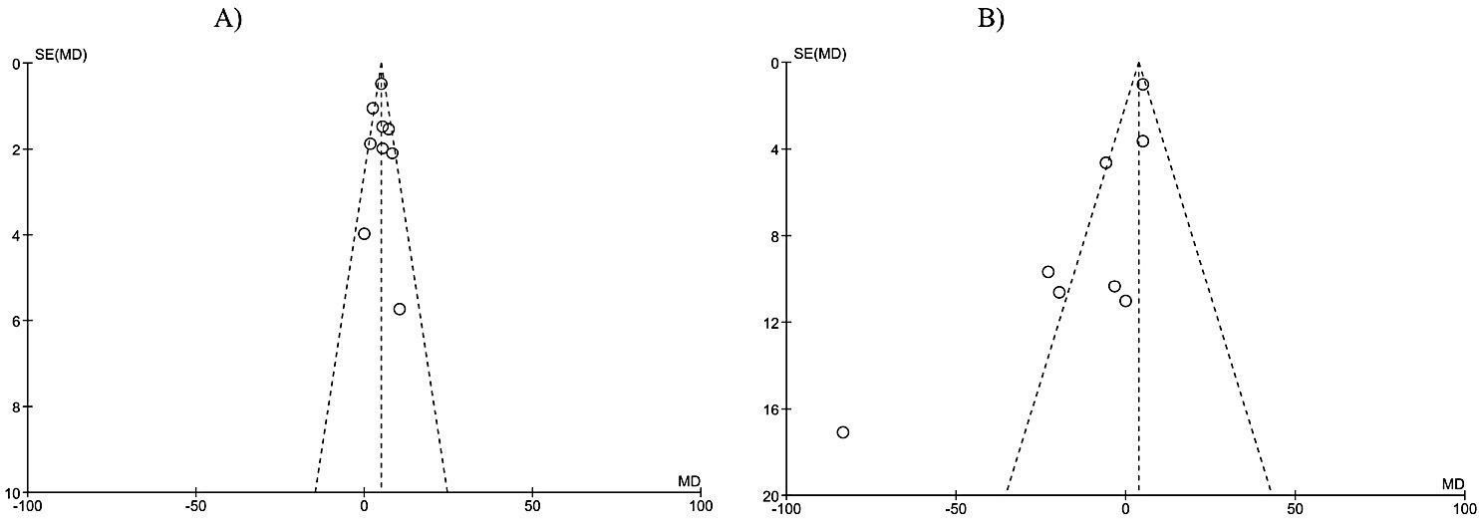
Funnel plot of studies included in the meta-analysis for the outcome of HDL (A) and LDL (B). RR = Relative Risk, SE = standard error. HDL= High Density Lipoprotein, LDL= Low Density Lipoprotein

The funnel plot test was conducted to evaluate potential publication bias of the present meta-analysis. In the present meta-analysis, we assessed the publication bias by examining funnel plot test of the effects of prebiotic/synbiotic on HDL and LDL. Symmetrical funnel plots suggested that there is no publication bias (Figure 3). The absence of publication bias was conﬁrmed by Egger’s linear regression of LDL (intercept: 1.5; standard error: 529; 95% CI: -11.4, 14.4; t= 0.28, df=6; two-tailed p= 0.78). Additionally, publication bias was not apparent by Begg’s rank correlation test (Kendall’s Tau with continuity correction: 0.03; z=0.12; two-tailed p= 0.9).

**Table 1:** Characteristics of included trials

**Auther**	**Journal**	**Year**	**Country**	**No. of Subjects in case group**	**No. of controls**	**Gender**	**Age (years)**	**Clinical Condition of Subjects**	**Follow-up Duration**	**Prebiotic or Synbiotic compounds**	**Dosage**	**Significant Outcome**	**Jadad score**
Asemi* et al.*	Clinical Nutrition	2014	Iran	31	31	F/M	35-70	T2DM	6 weeks	Synbiotic	Lactobacillus sporogenes (1 107 CFU), 0.04 g inulin (HPX) as prebiotic with 0.38 g isomalt, 0.36 g sorbitol and 0.05 g stevia as sweetener per 1 g, three times a day in a 9 g package	Reduction in serum insulin, FPG, triglyceride and hs-CRP; increase in HDL-C, total GSH and uric acid	3
Bonsu* et al.*	Int J Diabetes & Metab	2012	Canada	12	14	F/M	>40	T2DM	12 weeks	Prebiotic	10 g of inulin-based fiber/pelacpo:xylitol	No significant outcome	5
Dehghan* et al.*	Complementary Therapies in Medicine	2016	Iran	27	22	F	30-65	T2DM	2 months	Prebiotic	10 g of oligofructose-enriched inulin	Reduction in BMI,WC, HC, DBP, fasting serum glucose, HbA1c, serum lipids, IL-12, IFN-ϒ; increase in IL-4	5
Dehghan* et al.*	Int J Food Sci Nutr	2013	Iran	24	25	F	20-65	T2DM	8 weeks	Prebiotic	10g/d inulin/10g/d maltodextrin(placebo)	Reduction in FBS, HbA1c, total cholesterol, TG, LDL-C, LDL-C/HDL-C ratio, TC/HDL-C ratio; increase in HDL-c	5
Ebrahimi* et al.*	J diabetes metabolic disorders	2017	Iran	35	35	F M	58(average)	T2DM	9 weeks	Synbiotic	500 mg/day Streptococus thermophilus, Fructo oligosaccharide, 0.5 mg lactose	Improve the HbA1c, BMI and Microalbuminuria	4
Luo* et al.*	Human Nutrition and Metabolism	2000	France	10	10	F/M	57(average)	T2DM	4 weeks	Prebiotic	20 g/d Short-chain fructooligosaccharides (FOS)/ sucrose(placebo)	No significant outcome	1
Moroti* et al.*	Lipids Health Dis.	2012	Brazil	10	10	F	50-60	T2DM	4 weeks	Synbiotic	200 mL of a symbiotic shake containing 108 UFC/mL Lactobacillus acidophilus, 108 UFC/mL Bifidobacterium bifidum and 2 g oligofructose/placebo:200 ml of shake without synbiotic	Increase in HDL-c, reduction in fasting glycemia	2
Pourghassem* et al.*	Complementary Therapies in Medicine	2013	Iran	24	25	F	20-65	T2DM	8weeks	Prebiotic	10 g/day of inulin/ maltodextrin(placebo)	Reduction in FPG, HbA1c and MDA; increase in total antioxidant capacity and superoxide dismutase activity	5
Pourghassem* et al.*	Complementary Therapies in Medicine	2015	Iran	28	32	F	30-65	T2DM	8 weeks	Prebiotic	10g/d Resistant Starch (RS2)[2 packages of 5 g]	Reduction in HbA1c, TNF-α, triglyceride, increase in HDL-c	4
Shakeri* et al.*	Lipids	2014	Iran	26	26	F/M	35–70	T2DM	8 weeks	Synbiotic	40 g package of synbiotic bread or probiotic bread for a total of 120 g/day	Reduction in serum TAG, VLDL-C, TC/HDL-C; increase in HDL-c	5

### Subgroup analysis

As there is a significant heterogeneity among studies, we decided to explore the source of heterogeneity by subgroup analysis. Thus, we performed the analyses based on intervention (prebiotic or synbiotic) and study quality (high quality or low quality studies) (Table 2).

When analyzed based on intervention, TG, LDL and HDL showed a significant difference in synbiotic group compared with placebo (TG WMD = -44.94, 95% CI -80.96 to -8.92; LDL WMD = 4.8, 95% CI 2.77 to 6.84; HDL WMD = 4.94, 95% CI 4.05 to 5.83). In regard to second type of intervention, prebiotic group had significant decrease in TG (WMD= -31.29, 95%CI -49.85 to -12.73), TC (WMD= -15.01, 95%CI -27.44 to -2.58), FBG ( WMD= -12.40, 95%CI -15.86 to -8.94) and HbA1c (WMD=-0.47, 95%CI -0.8 to -0.13), marginally significant decrease in LDL (WMD= -20.78, 95%CI -43.96 to 2.40) and significant increase in HDL (WMD= 4.75, 95%CI 3.39 to 6.12)(Table 2). Another subgroup analysis was performed considering study quality. In high quality studies, TG, TC, FBG and HbA1c were decreased significantly in intervention group in comparison with control group (WMD=-37, 95%CI -51.82 to -22.19 for TG, WMD= -13.17, 95%CI -21.42 to -4.93 for TC; WMD=- 13.66, 95% CI -15.94 to -11.39 for FBG; WMD= -0.55, 95%CI -0.76 to -0.33 for HbA1c). Moreover in this subgroup, HDL showed a significant increase in intervention group compared with placebo (WMD= 5.41, 95% CI 3.91 to 6.92). Low quality studies showed significant increase in HDL (WMD= 4.72, 95%CI 3.86 to 5.58) and LDL (WMD= 5.09, 95%CI 3.08 to 7.11) while other factors had non-significant changes in this subgroup (Table 2).

**Table 2:** Subgroup analysis*

**Subgroup**		**TG**			**TC**			**LDL**		
		**WMD** **(95% CI)**	**Test for heterogeneity (I2, P)**	**overall effect** **(P value)**	**WMD** **(95% CI)**	**Test for heterogeneity (I2, P)**	**overall effect** **(P value)**	**WMD** **(95% CI)**	**Test for heterogeneity (I2, P)**	**overall effect** **(P value)**
**Intervention**	Synbiotic	-44.94 [-80.96, -8.92]	59%, p=0.09	p=0.01	-6.18 [-26.11, 13.75]	87%, p<0.0001	p=0.54	4.80 [2.77, 6.84]	0%, p=0.5	p<0.001
	Prebiotic	-31.29 [-49.85, -12.73]	47%, p=0.11	p=0.001	-15.01 [-27.44, -2.58]	33%, p=0.20	p=0.02	-20.78 [-43.96, 2.40]	88%, p<0.001	p=0.08
**Quality of Study**	Low Quality	-11.26 [-49.43, 26.90]	97%, p<0.001	p=0.56	-3.59 [-31.65, 24.46]	84%, p=0.002	p=0.8	5.09 [3.08, 7.11]	0%, p=0.99	p<0.001
	High Quality	-37.00 [-51.82, -22.19]	6%, p=0.38	p<0.001	-13.17 [-21.42, -4.93]	6%, p=0.38	p=0.002	-18.06 [-35.40, -0.71]	81%, p=0.0001	p=0.04
**Subgroup**		**HDL**			**FBG**			**HbA1c**		
		**WMD (95% CI)**	**Test for heterogeneity (I2, P)**	**overall effect** **(P value)**	**WMD** **(95% CI)**	**Test for heterogeneity (I2, P)**	**overall effect** **(P value)**	**WMD (95% CI)**	**Test for heterogeneity (I2, P)**	**overall effect** **(P value)**
**Intervention**	Synbiotic	4.94 [4.05, 5.83]	26%, p=0.26	p<0.0001	-3.33 [-56.83, 30.16]	99%, p<0.001	P=0.55	Not applicable	Not applicable	Not applicable
	Prebiotic	4.75 [3.39, 6.12]	64%, p=0.03	p<0.0001	-12.40 [-15.86, -8.94]	68%, p=0.009	p<0.0001	-0.47 [-0.8, -0.13]	92%, p<0.001	p<0.0001
**Quality of study**	Low Quality	4.72 [3.86, 5.58]	62%, p=0.07	p<0.001	-12.95 [-45.79, 19.89]	99%, p<0.001	p=0.44	Not applicable	Not applicable	Not applicable
	High Quality	5.41 [3.91,6.92]	46%, p=0.1	p<0.001	-13.66 [-15.94, -11.39]	27%, p=0.21	p<0.001	-0.55 [-0.76, -0.33]	90%, p<0.001	p<0.001

*: Abbreviations: TG, triglycerides; TC, total cholesterol; LDL-C, low-density lipoprotein cholesterol; HDL-C, high-density lipoprotein cholesterol;; FBG, fasting blood glucose; WMD, weighted mean difference; CI, confidence interval.

The heterogeneity was decreased significantly after subgroup analysis especially for study quality subgroup. In subgroup analysis based on intervention, the prebiotic and synbiotic group showed no significant heterogeneity across the trials in regard to TG/cholesterol and TG/LDL/HDL respectively (Table 2). On the other hand, subgroup analysis by study quality, showed the most reductive effect on heterogeneity. It has been shown that except for LDL and HbA1c, there is no significant heterogeneity across the trials in regard to other factors (Table 2).

Vulevic et al reported that a galactooligosaccharide mixture could reduce markers of metabolic syndrome and modulate immune function in overweight adults.^34^ A pilot study demonstrated that prebiotic consumption might beneficially affect insulin level, with no significant effects on plasma lipids, in patients with non-alcoholic steatohepatitis.^35^ Eslamparast and her colleagues reported that synbiotic supplement can help in the management of metabolic syndrome and insulin resistance.^36^ Two other studies also suggested protective effects of prebiotics in patients with prediabetes.^13,37^ Published meta-analyses in this area are limited in number.

A recent meta-analysis has been conducted on the effects of prebiotics on glycaemia, insulin concentrations and lipid parameters in overweight and obese adults and the results showed positive effects of prebiotics and synbiotics on dyslipidemia and insulin resistance.^32^ Another systematic review was conducted to evaluate metabolic benefits of prebiotics in human subjects. The results indicated that prebiotic consumption is associated with improved self- reported feelings of satiety along with reduced postprandial glucose and insulin concentrations.^38^ To the best of our knowledge, the present study is first to systematically evaluate effects of prebiotic consumption on glycaemia and lipid profile in T2DM patients. In the present study, a significant heterogeneity was found among individual studies for target indicators (except for HDL-c).Two subgroup analyses were conducted based on intervention type (prebiotic or synbiotic), and study quality (high quality vs. low quality studies).

After the subgroup analysis by intervention, the prebiotic subgroup showed no heterogeneity in TG and TC significantly. The heterogeneity in TG, LDL and HDL has been removed after the subgroup analysis based on the synbiotic intervention. Anyway, the quality of studies was shown as the most important source of heterogeneity. The heterogeneity of all outcomes, except for LDL and HbA1C, was removed after the subgroup analysis of seven high quality studies.^16,22-25,27,31^ Thus, we can assume that the source of heterogeneity is partially related to quality of the studies.

Based on intervention, synbiotic consumption led to significant improvements in TG, HDL-c, and LDL-c concentrations. Other intervention, prebiotic, significantly improved TC, TG, FBG, and HbA1c.

When analyzed by study quality, high quality studies showed beneficial effects of prebiotic/synbiotics on factors of glycaemia and lipid markers (except for LDL-c), while in low quality studies, intervention group had only significant improvements in HDL-c.

There are controversial results on the efficacy of prebiotics/synbiotics in improvements of lipid profile and glycemic index. Increasing enteroendocrine cell activity, improved glucose homeostasis and modulated gut microbiota by intake of prebiotics, especially FOS, have been shown via prior studies.^39,40^ On the other hand, some studies could not find these favorable effects of prebiotics; they showed no significant effects on glycemic and lipid indices, especially lipid profiles, in diabetic participants.^41,42^ These controversial findings, and of course, the significant heterogeneity reported for our included studies, might be a result of different probiotic strains and prebiotic types, administration dosage, clinical characteristics of participants, duration of intervention, or lack of appropriate controls or placebo.^43^

Our study is supportive of the idea that prebiotic/synbiotic consumption contribute to positive effects on blood lipid fractions; several mechanisms are proposed explaining this relationship. Inulin- type fructans reduce the *denovo* synthesis of fatty acids in the liver, thus result in decreased levels of serum or liver TG.^44^ The bacterial fermentation of non-digestible oligosaccharides (NDOs) in GI tract, leads to the formation of short chain fatty acids (SCFAs) including propionate, butyrate and acetate with different ratios depending on the substrate type.^45^ 3-hydroxy-3-glutaryl-Co-A (HMG CoA) reductase, is a key enzyme in cholesterol synthesis; by inhibiting its activity, propionate might play a role in serum cholesterol reduction.^46^ Probiotics can also reduce intestinal cholesterol absorption accompanied by its increased fecal excretion.^43^

In this meta-analysis prebiotic/synbiotics showed promising effects in glucose homeostasis. Studies have explained the underlying mechanisms: soluble fibers can delay gastric emptying, retard entry of glucose into blood stream, and decrease the postprandial rise of serum glucose. In addition, soluble fibers modify the secretion of GLP-1 that is a gut hormone engaged in glucose metabolism; they also lead to SCFA production and therefore may affect serum glucose and insulin levels.^27^ On the whole, probiotics and prebiotics are safe products. However, high doses of prebiotics increase the risk of bloating, flatulence and GI discomfort which might widely vary from person to person depending on the type of food.^47^

Our study encounters some basic limitations. Using Q statistics and I_2_, the included studies showed significant heterogeneity. Subgroup analyses were conducted to detect the source of heterogeneity. However, such heterogeneity still remained in most subgroups, except for quality of studies. One limitation of the meta-analysis is that some of the studies included in the meta-analysis are not independent. Seven studies of ten studies are from the same country (Iran). They are different publications, but the data seem to originate from the same groups of subjects. Another limitation of the present meta-analysis is the fact that there are no included trials with T1DM patients. Therefore, the findings and their interpretations are limited to T2DM patients.

Clinical heterogeneity between studies can lead to statistical heterogeneity in their results. In addition, this meta-analysis indicated possible publication bias in LDL but not in HDL. It is maybe because we included the studies, which were conducted with the same population (country and geographical region).

Publication bias has been reported in several large meta-analyses published in major medical journals; significant and positive results are more probable to be published and this is the main reason for such reported bias. Our meta-analysis included some methodologically low quality studies, which is another key source of bias. Since smaller studies need larger treatment effects to be published, they are more prone to such noted biases.

In subgroup analyses conducted based on study quality, stronger beneficial effects were found in treatment group in comparison with control one. Based on this finding, we can conclude that either heterogeneity or true treatment effect could be the cause of publication bias.

## Conclusion

Conclusively, our meta-analysis found that diets supplemented with either prebiotics or synbiotics can result in improvements in lipid metabolism and glucose homeostasis in patients with T2DM. Even though the overall analysis did not show significant changes for TC and LDL-c, subgroup analyses could find more noticeable changes in these markers.

Considering the limitations for individual trials, prebiotics/synbiotics cannot be prescribed as alternative medicine T2DM, but these patients might benefit from these components as a complementary advise besides medicine and lifestyle modifications.

More research are suggested with larger sample sizes, to determine the effective and also safe dose, duration and the best combinations of probiotics and prebiotics to reach a maximum positive effect.

## Ethical Issues

Not applicable.

## Conflict of Interest

No potential conflict of interest relevant to this article was reported.
